# Within-Person Variation in Ultra-Processed Food Consumption Is Associated with Total Daily Energy Intake

**DOI:** 10.3390/nu18132075

**Published:** 2026-06-24

**Authors:** Maria Fernanda Gombi Vaca, Euridice Martinez-Steele, Giovanna Calixto Andrade, Maria Laura da Costa Louzada, Renata Bertazzi Levy

**Affiliations:** 1 Rudd Center for Food Policy and Health, University of Connecticut, One Constitution Plaza, Suite 600, Hartford, CT 06103, USA; 2 Department of Preventive Medicine, School of Medicine (FMUSP), University of São Paulo, Av. Dr. Arnaldo, 455, São Paulo 01246-903, SP, Brazil; 3 Center for Epidemiological Research in Nutrition and Health (NUPENS), University of São Paulo, Av. Dr. Arnaldo, 715, São Paulo 01246-904, SP, Brazil; 4 Department of Nutrition, School of Public Health, University of São Paulo, Av. Dr. Arnaldo, 715, São Paulo 01246-904, SP, Brazil

**Keywords:** ultra-processed food, energy intake, within-person, dietary survey, ad libitum food intake, energy compensation

## Abstract

Background/Objectives: Important gaps remain in understanding the association between ultra-processed food (UPF) consumption and same-day energy intake. This study aimed to expand the understanding of how within-person variation in UPF consumption is associated with total energy intake. Methods: Nationwide, representative dietary survey data from 38,854 participants (≥10 years old) in the 2017–2018 Brazilian Household Budget Survey food intake module were analyzed. These cross-sectional repeated-measures data were used to estimate within-person differences in energy intake between two days of food consumption. Mixed models were applied to examine the associations between the presence and energy share of UPFs in the diet and total daily energy intake. Results: Among participants who consumed UPFs on only one of the two days (*n* = 8055), daily energy intake was higher on the day when UPFs were consumed than on the day when UPFs were not consumed (1699 kcal vs. 1530 kcal; *p* < 0.001). On average, among all participants (*n* = 38,854), a 10 percentage-point increase in UPF energy share on a given day was associated with an increase of 39 kcal in energy intake over the day. We found statistically significant effect modification by sex (*p* = 0.024) and age group (*p* < 0.001). Supplementary analyses suggest that the association between UPF energy share and total energy intake is consistent with partial replacement of non-UPF by UPF and is unlikely to reflect differences in food quantity or energy density. Conclusions: Among participants who consumed UPFs on only one of the two days, the day with UPF consumption was associated with higher total daily energy intake than the day without UPF consumption. A higher within-person UPF energy share was also associated with higher total daily energy intake. Understanding the association between UPF consumption and same-day energy intake can inform public health strategies aimed at reducing excessive energy intake.

## 1. Introduction

Ultra-processed foods (UPFs) are industrially formulated products that often combine additives [1] not found in a typical home kitchen and are designed for hyper-palatability and extended shelf life [2,3]. The consumption of UPF has been associated with weight gain [4,5], an increased risk of obesity [4,6,7], as well as with several non-communicable diseases, such as cardiovascular and metabolic diseases, diabetes, depression, cancer, and all-cause mortality [8,9,10].

It has been hypothesized that the role of UPFs in diet-related diseases is due to UPFs’ nutrient profile. UPFs are generally energy-dense, low in essential nutrients, and high in added sugar, sodium, and fat, all of which are key contributors to various noncommunicable diseases [2,3,11,12]. Beyond nutrient composition, UPFs are posited to promote excess energy intake through mechanisms that include rapid eating rates [5,13], low satiety [14,15], and the use of flavor enhancers and cosmetic additives [1] that may disrupt appetite regulation [16,17,18]. Observational and short-term experimental studies have found that diets high in UPFs are associated with higher energy intake [5,19,20,21], yet important gaps remain in understanding the immediate association between UPF consumption and same-day energy intake.

In this study, we seek to further understand how within-person variation in UPF consumption translates into total energy intake by quantifying the energy intake associated with the energy share of UPFs in a non-experimental, free-living (i.e., individuals are engaged in their usual daily activities and face real-world constraints, such as food prices, access, and availability) and ad libitum (i.e., individuals’ food consumption is not restricted or controlled) setting. Specifically, using two days of detailed dietary intake data from a nationally representative sample, we aimed to assess whether the same person consumes more total energy on days when they eat a higher share of UPFs than on days when they eat a lower share. This within-person, real-world approach can provide novel insights into same-day energy compensation in relation to UPF consumption.

## 2. Methods

### 2.1. Study Population

We conducted a secondary analysis of a nationally representative sub-sample of participants in the 2017–2018 Brazilian Household Budget Survey (BHBS) [22,23]. The master sample of household participants in the BHBS was obtained by a two-stage complex cluster sampling design. In the first stage of selection, primary sampling units were 5404 census tracts selected by proportional probability to the number of households in the strata. Census tracts were previously obtained by geographic and income stratification based on the 2010 Brazilian Demographic Census. In the second stage of selection, sampling units were households selected by simple random sampling. Then, a sub-sample of 20,112 households was selected from the master sample by simple random sampling [22]. In the sub-sample, a total of 46,164 individuals aged 10 years or older responded to the food intake module survey and self-reported sociodemographic information to a trained interviewer [22]. In this secondary analysis, participants who completed two 24-h dietary recalls on non-consecutive days within the same week, *n* = 38,854 (84.2% of the respondents in the sub-sample), were included. Data collection occurred between 11 July 2017 and 9 July 2018.

### 2.2. Food Intake Data Collection and Specific Procedures

Dietary data were collected using 24 -hour dietary recalls following a structured protocol based on the Multiple Pass 24-h Recall [24], and information was recorded using a tablet and software designed specifically for this survey [22]. Participants self-reported all types of food and beverages consumed over the previous 24 h (hourly, from 12 a.m. to 11 p.m.). Dietary information included the amount of each food or beverage consumed (in grams), the cooking method, where applicable, the day of the week, the location, and the self-reported type of eating occasion [22].

The energy content in calories (kcal) and the amount of total carbohydrate (g), protein (g), fat (g), fiber (g), and sodium (mg) of each food and beverage consumed were estimated using the “TBCA—Nutritional composition table of food consumed in Brazil”, version 7.1 [25]. Detailed pre-test, validation, and quality control procedures adopted during data collection are available elsewhere [22,26].

### 2.3. Diet-Related Variables

For this study, we classified each food and beverage consumed according to the Nova food classification system, which categorizes foods by the degree and extent of processing [2]. Foods were categorized into one of two groups: (1) non-UPF, including three Nova food groups (unprocessed or minimally processed food; processed culinary ingredient; and processed food), or (2) UPF, characterized by food and beverages that are the result of an intensive degree of processing with additions of substances of no or rare culinary use. Examples of non-UPFs include foods primarily associated with the traditional dietary pattern in Brazil, such as fruits, vegetables, bakery breads, and “home-made” culinary preparations (e.g., rice and beans, pasta with sauce, beef or chicken dishes). Examples of UPFs are sweet and savory snacks, carbonated soft drinks, sweetened dairy drinks, breakfast cereals, and frozen meals, among others [2]. The grouping of non-UPF categories was intended to contrast foods characterized by industrial formulations and cosmetic additives with those that more closely reflect traditional food preparation practices. In the Brazilian context, minimally processed foods, processed culinary ingredients, and most processed foods (classified as non-UPF in the study) are more closely aligned with traditional dietary patterns centered on home-prepared meals, and therefore were grouped to represent the contrast to UPF consumption. Because detailed preparation-level information was not available for all items, certain foods (e.g., pizzas, cakes, mixed dishes, and fruit drinks) with unclear Nova group classification were classified based on the most common form of consumption in Brazil or based on the predominant ingredient or preparation method. For instance, “pizzas” are typically ready-to-heat products and were, therefore, classified as UPFs. “Cakes” and “mixed dishes” were classified as UPF if they contained a UPF component (e.g., chocolate, mayonnaise). “Fruit drinks” were assumed to be industrialized and UPF, but were assumed to be non-UPF if they were specified as “fresh/natural”. Additional details and classification rules for commonly ambiguous items are provided in Supplementary Table S1. UPFs were classified into subgroups for descriptive purposes.

Each day of the participant’s 24-h dietary recall was categorized according to the presence of UPFs (binary variable) as a “day without UPF” or “day with UPF”. Next, participants were categorized according to their UPF consumption pattern as one of the following: (1) “No UPF consumption on any day”; (2) “UPF consumption on one day only”; or (3) “UPF consumption on both days”. Participants categorized in the latter two patterns (categories 2 and 3) were categorized as “UPF consumers”.

We obtained each participant’s total energy intake (kcal), total amount of food consumed (g), and UPF energy share (% of total calories; % kcal) for each day. We calculated the energy density for each day by dividing total energy intake (kcal) by the total amount of food consumed (g), based on all foods and beverages (including water) consumed during the day. We obtained nutrient densities of total carbohydrate, protein, fat, and fiber in grams (g) per 1000 kcal, and of sodium, in milligrams (mg) per 1000 kcal, for each day.

### 2.4. Sociodemographic Variables

Participants’ characteristics analyzed in the study were as follows: sex (male or female), age group (adolescents, 10–19; adults, 20–59; elders, 60 years or older), and schooling (4 years or less, 5–8 years, 9–12 years, or 13 years or more), household per capita income (calculated by dividing self-reported household income in Brazilian Reais, R$, by the number of individuals living in the household), geographic area (rural or urban), and geopolitical region (North, Northeast, Southeast, South and Center–West).

### 2.5. Data Analysis

We calculated the weighted averages of total daily energy intake and daily UPF energy share over the two days, and the proportion of UPF consumers in the sample (i.e., those consuming UPFs at least once over the two days). We then compared these values by sociodemographic characteristics using univariate regression on survey data. We applied the Bonferroni adjustment for multiple comparisons to the non-ordinal variables (sex, geographic area, and geopolitical regions), and the linear trend test to the ordinal variables (age group, schooling, and income).

To estimate the total energy intake on a given day (kcal; dependent variable) associated with the presence of UPFs on that day (independent variable), we fit a mixed model with an interaction term between the presence of UPFs in the day (“day without UPF” or “day with UPF”) and UPF consumption pattern (“No UPF consumption on any day”; “UPF consumption on one day only”; or “UPF consumption on both days”). We compared total energy intake between days of the UPF patterns using a z-test and Bonferroni adjustment for multiple comparisons of the marginal estimates from the model. Average energy and nutrient densities for each day of the UPF consumption patterns were described. Total energy intake on each day for participants in the “UPF consumption on one day only” pattern was described. Average energy intake by UPF subgroups on the day with UPF in the “UPF consumption on one day only” pattern was described by sex and age group.

Next, we fit a second mixed model to assess the association between total energy intake (kcal; dependent variable) and UPF energy share (% kcal; independent variable). In this model, we estimated both the within- and between-person effects of the energy share of UPF on energy intake. To estimate both effects, the independent variable UPF energy share was decomposed into within-person and between-person components. The between-person variable represented the participant’s mean UPF energy share (${\bar{X}}_{p})$ across the two days, and the within-person variable represented the daily UPF energy share’s deviation from the participant’s two-day mean UPF energy share ($X_{pd}-{\bar{X}}_{p}$). The within- and between-person estimates were formally tested for equality using a test for linear combinations of coefficients. When within- and between-person estimates are not equal, the within-person coefficient is generally less prone to confounding by time-invariant individual characteristics under the model assumptions [27,28]. In other words, all stable characteristics of the participants, observed or not, are controlled for because only within-person variation is used to estimate within-person effects, meaning that each individual truly acts as their own control [27,28]. Therefore, the model formula was specified as:

$$Y_{pd}\;=\;\beta_{0}\;+\;\mu_{p}\;+\;\beta_{1}^{W}\left(X_{pd}\;-\;{\bar{X}}_{p}\right)\;+\;\beta_{2}^{B}{\bar{X}}_{p}\;+\;\epsilon_{pd}$$

whereas $\left(X_{pd}\;-\;{\bar{X}}_{p}\right)$ represents the within-person variable and $\beta_{1}^{W}$ represents the within-person estimate, and ${\bar{X}}_{p}$ represents the between-person variable and $\beta_{2}^{B}$ represents the between-person estimate.

All mixed models included a random intercept for participants ($\;\mu_{p}$) to account for repeated observations (i.e., daily food consumption) nested within participants, capture within-person correlation, and allow participants to differ in their average starting level of the outcome, daily energy intake. The mixed models were adjusted for the time-variant covariates and participant-level time-invariant covariates. The time-variant covariates were day of the week (weekday or weekend) and location (at home or away from home), both representing contextual time-varying factors that might influence energy intake [29,30]. In the models, an “away from home” location refers to a day when at least one of the reported eating occasions was not consumed at home. Individual-level time-invariant covariates included in the adjusted models were sex, age group, schooling, household per capita income, geographic area, and geopolitical region. The time-invariant covariates were included to adjust for the between-person effect estimation. We tested for effect modification by sex and age in the model assessing the association between the daily energy share of UPF and energy intake. We examined model residuals for normality and constant variance. To assess the stability of the findings, we conducted a robustness check restricted to participants in the “UPF consumption on one day only” group using a paired *t*-test to compare total daily energy intake on the day without UPF consumption versus the day with UPF consumption. We conducted sensitivity analyses to better understand the part-whole relationship between UPF energy share and total energy intake, and to clarify whether the observed association was consistent with energy addition or replacement. Using the same model specifications, we tested energy intake from UPFs and energy intake from non-UPFs as two separate dependent variables. We conducted an additional sensitivity analysis by excluding foods with the most uncertain classification (i.e., the UPF subgroups “ready-to-heat pizza”, “cakes and pies”, “mixed dishes”, and “fruit drinks”) to test whether the within-person coefficient remained robust and to explore potential misclassification bias. 

In addition to the sensitivity analyses, we conducted supplementary analyses to better contextualize the relationship between UPF energy share and total energy intake. Specifically, using the same primary mixed model specifications, we estimated the association between UPF and non-UPF energy intake (independent variables) and total daily energy intake (dependent variable). In addition, we estimated a model with UPF energy intake as the dependent variable and non-UPF energy intake as the independent variable to examine the extent to which UPF intake is associated with changes in non-UPF intake. These analyses were intended to provide additional context on dietary composition and how one dietary component changes relative to the other. We also estimated the association between UPF energy share (independent variable) and alternative dietary measures, namely, energy density (kcal/g) and total food consumed (g) (dependent variables). These analyses were exploratory and were not intended to establish causal relationships, but rather to provide additional insight into potential mechanistic interpretation.

The mixed-effects models did not incorporate the complex sampling design. The level of statistical significance was set at 5%. All statistical analyses were conducted using Stata 18 (version 18.5).

## 3. Results

The sociodemographic characteristics and diet-related variables of the study population are presented in Table 1. The average daily energy intake was 1753 kcal (Standard Error [SE] = 8), and the average UPF energy share was 20.6% (SE = 0.2). The weighted proportion of participants who consumed UPF on at least one day was 92.6% (SE = 0.2). whereas the unweighted proportion was 90.5% (Supplementary Figure S1). Daily energy intake was higher among male participants, and the frequency of UPF consumers and the UPF energy share were higher among female participants. Daily energy intake and the frequency of UPF consumers, and the average UPF energy share increased with schooling and income, and decreased with age.

Supplementary Figure S1 shows the predicted adjusted mean energy intake on each day associated with the presence of UPF in the diet across all three UPF consumption patterns. Comparisons across the three patterns reflect between-person differences in average intake relative to UPF consumption on a given day. The “UPF consumption on one day only” pattern permits a within-person comparison, which showed higher energy intake on the day with UPF than on the day without UPF. Supplementary Table S2 provides the average energy and nutrient density on each day, descriptively, by UPF consumption pattern. Across all participants, the unweighted mean within-person difference between the two days in UPF energy intake was 287 kcal (SE = 2.0; 95% CI, 284 to 291) and in non-UPF energy intake was 501 kcal (SE = 2.6; 95% CI, 496 to 506). 

**Figure 1:**
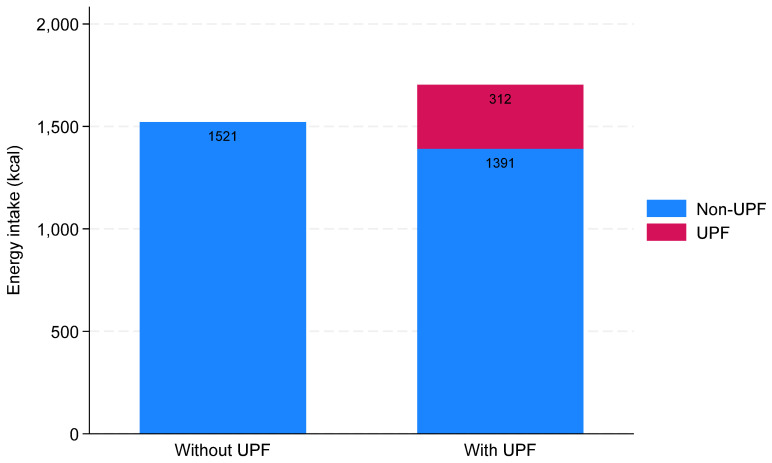
Unweighted observed total energy intake (kcal) on each day for participants in the “ultra-processed food (UPF) on one day only” pattern (Brazil, 2017-2018, *n* = 8055). Abbreviation. UPF: ultra-processed food. Note. Using a paired *t*-test, we found that total daily energy intake was 181 kcal higher (95% CI, 164 to 199; *p* < 0.001) on the day with UPF consumption than on the day without UPF consumption.

Figure 1 shows the unweighted observed total energy intake on each day for participants in the “UPF consumption on one day only” pattern, representing a within-person comparison. On the day without UPF, mean total energy intake was 1521 kcal (SE = 8.2), and on the day with UPF, mean total energy intake was 1702 kcal (SE = 9.4) Supplementary Table S3 describes the unweighted observed average energy intake (kcal) from UPF subgroups on the day with UPF among participants in the “UPF consumption on one day only” pattern, by sex and age group.

**Figure 2:**
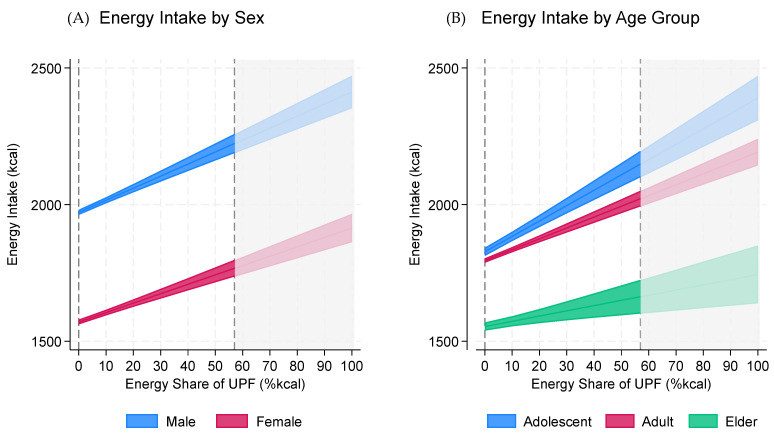
Adjusted mixed model predictive daily energy intake (kcal; mean and 95% CI) across levels of energy share of ultra-processed foods (UPFs) by sex (**A**) and by age group (**B**) (Brazil, 2017–2018, N = 38,854). Abbreviation. UPF: ultra-processed food. Note. The mixed model was adjusted by day of the week, location, sex, age group, schooling, per capita income, geographic area, and geopolitical region. Dashed vertical lines indicate the 5th and 95th percentiles of the observed UPF energy share distribution. Values outside this range (in lighter color) should be interpreted as extrapolations.

The results from unadjusted and adjusted mixed models testing the association between the energy share of UPF and total energy intake are presented in Table 2. The unadjusted intraclass correlation coefficient was 0.514 (95% CI, 0.506 to 0.521), indicating moderate clustering. We found that the within-person and between-person estimates were not equal in the model (*p* < 0.001). Therefore, we used the within-person estimate to interpret the association. The adjusted within-person coefficient obtained was 3.90 (95% CI, 3.50 to 4.29, *p* < 0.001), meaning that, on average, an increase of 10 percentage points in the energy share of UPF of a given person was associated with an increase of 39 kcal in energy intake over a day for that person. In exploratory effect modification analyses, we observed evidence of heterogeneity by sex (males: reference group; females: β = −0.88, 95% CI, −1.68 to −0.12, *p* = 0.024) and by age group (adolescents: reference group; adults: β = −1.60, 95% CI, −2.54 to −0.67, *p* = 0.001; elders: β = −3.67, 95% CI, −4.99 to −2.35, *p* < 0.001), without adjustment for multiple interaction tests. The adjusted within-person estimate for female participants was 3.45 (95% CI, 2.93 to 3.97, *p* < 0.001), and for male participants was 4.42 (95% CI, 3.82 to 5.01, *p* < 0.001). The adjusted within-person estimate for adolescents was 5.63 (95% CI, 4.83 to 6.44, *p* < 0.001); for adults, 3.98 (95% CI, 3.49 to 4.47, *p* < 0.001); and for elders, 1.91 (95% CI, 0.86 to 2.96, *p* < 0.001). Figure 2 presents the predictive values for daily energy intake from the adjusted models, stratified by sex (Figure 2A) and age group (Figure 2B), according to the energy share of UPF. Supplementary Figure S2 shows the observed distribution of UPF energy share by sex and age group as a reference for interpreting the predictive values in Figure 2.

In the robustness check restricted to participants in the “UPF consumption on one day only” group using a paired *t*-test, we found that total daily energy intake was 181 kcal higher (95% CI, 164 to 199; *p* < 0.001) on the day with UPF consumption than on the day without UPF consumption. This result is consistent with the findings in the main analysis and supports the robustness of the observed within-person association. In sensitivity analyses, each one percentage-point increase in UPF energy share was associated with 19.2 kcal (β = 19.2, 95% CI 19.0 to 19.3, *p* < 0.001) higher energy intake from UPFs and 15.3 kcal (β = −15.3, 95% CI −15.6 to −15.0, *p* < 0.001) lower energy intake from non-UPFs. This sensitivity analysis is consistent with, but does not prove that a higher UPF energy share reflects both higher UPF energy intake and a partial displacement of non-UPF calories, with incomplete overall energy compensation for UPFs. The results of the sensitivity analysis that excluded the most uncertain classification (i.e., UPF subgroups “ready-to-heat pizza”, “cakes and pies”, “mixed dishes”, and “fruit drinks”) are presented in Supplementary Table S4. We found that the within-person coefficients were slightly smaller than the coefficient from the main analysis. These sensitivity analyses suggest that potential misclassification would have left the estimates generally unchanged in direction and magnitude relative to the primary findings.

Supplementary Table S5 presents alternative models to provide additional context on the relationships among dietary components. In the model examining the association between UPF and non-UPF energy intake and total energy intake, we observed that each additional 1 kcal from UPF was associated with a 0.763 kcal increase in total energy intake, while each additional 1 kcal from non-UPF foods was associated with a 0.889 kcal increase in total energy intake. Coefficients below one suggest that increases in a given energy source are associated with partial compensation of other components, such that total intake increases by less than the full amount, reflecting the mathematical structure of total energy intake, and should be interpreted descriptively. In the model examining the association between UPF energy intake and non-UPF energy intake (Supplementary Table S5), we observed that higher UPF energy intake was associated with lower non-UPF energy intake (β = −0.237; *p* < 0.001), which is consistent with, but does not demonstrate, partial substitution of non-UPF foods by UPF. In supplementary models examining the association between UPF energy share and alternative dietary measures (Supplementary Table S5), we observed that UPF energy share showed little to no association with energy density (β = 0.003; *p* < 0.001) and was not significantly associated with total food consumption (*p* = 0.462). These supplementary analyses provide limited support for mechanistic explanations and suggest that differences in total energy intake associated with UPF energy share are unlikely to be primarily associated with changes in energy density or food volume.

## 4. Discussion

In our study, we observed that an increased UPF energy share in an ad libitum, free-living food-consumption setting was associated with higher energy intake. The within-person variation in energy intake relative to the energy share of UPF in the diet in a large, nationally representative sample of individuals further elucidated the association of UPFs with same-day food intake. This study found that, when comparing the same person’s dietary intake over two days, energy intake on the day without UPF was lower than on the day with UPF. We also found that, in a between-person comparison, participants in the “UPF consumption on both days” pattern had higher energy intake than those who consumed UPF on one day only, on the days when UPF was consumed, and higher energy intake than those in the “no UPF consumption on any day” pattern, when UPF was not consumed. This finding suggests a potential explanation for the evolving literature that supports the association between UPFs and higher energy intake [5,20,21], and is consistent with the evidence that cumulative intake of UPFs can lead to weight gain [4,5].

The design of our study is parallel to controlled studies that assess the association of specific foods with energy intake. In such studies, energy compensation is estimated by comparing energy intake between a scenario with the consumption of a test food and a scenario without it, using a preload paradigm [31,32]. In our observational study, the within-person comparison of energy intake across two days in a free-living setting revealed the association between UPF-containing dietary days and higher same-day total energy intake. In our model, UPF energy share (independent variable) is defined relative to total daily energy intake (dependent variable), and its association with total energy intake is influenced by a part-whole relationship. Sensitivity and supplementary analyses showed that higher UPF energy intake was associated with lower non-UPF energy intake, suggesting that the association between UPF consumption and total energy intake was compatible with partial replacement of non-UPF with UPF, and a pattern of incomplete same-day energy compensation. However, because UPF and non-UPF energy intake are compositional and mathematically interdependent, this relationship reflects both potential behavioral patterns and structural constraints of the data. Our cross-sectional, repeated-measures study design cannot explicitly indicate whether UPFs were consumed in addition to, or in place of, non-UPFs, nor can it demonstrate the temporal sequence between UPF consumption and other foods, or that UPF consumption causes higher energy intake.

Using a similar observational study design, a previous study measured the potential for energy compensation from sugary beverages, including ultra-processed soda and fruit drinks [33]. In the study, increased energy intake was associated with sugary beverage consumption, which could be compatible with reduced energy compensation for these beverages over a day. The study suggested that higher energy intake may be due to the low satiety potential of sugary beverages, likely driven by their liquid form, which is rapidly available to the body, rather than by an equivalent amount of energy consumed in a solid form. Likewise, our study’s finding that UPF consumption is associated with higher energy intake within the same individual suggests possible explanations related to UPFs’ properties. UPFs have attributes such as their nutrient content, linked to high energy density [11,12], hyperpalatability, and texture and sensory characteristics that facilitate increased chewing rates [13]. These UPFs’ attributes can also influence gut processes that regulate appetite, food intake, and satiety [14,15]. These mechanisms partially explain UPFs’ short-term association with higher total daily energy intake [16,17,34]. However, these mechanisms and pathways were not directly measured in our study.

In supplementary analyses, we observed a small, near-null association between UPF energy share and energy density, and no significant association with total food consumption. These findings are consistent with the interpretation that a higher UPF energy share is not primarily related to differences in food volume or energy density. Although these supplementary analyses were intended to provide additional mechanistic context, they offer limited explanatory insight and do not support causal inferences.

We found differences in the association between UPF energy share and energy intake by sex and by age group. This association was slightly stronger in male than female participants and was stronger among adolescents than among adults and elders. Our study cannot determine whether the differences by sex and age group are due to variations in hormones, satiety, appetite, food preferences, eating occasions, or social context [35,36,37]. However, our descriptive analysis of UPF subgroups consumed among participants in the “UPF consumption on only one day” pattern suggests that snack-like foods (e.g., savory snacks, sweets, cookies and pastries, and soft drinks) are among the UPF subgroups contributing the greatest mean energy intake from UPF on that day. Prior studies observed that adolescents’ consumption of sugar-sweetened beverages and ultra-processed snack-type foods is higher than that of adults or elders [38,39,40]. Previous research suggests little or no energy-intake compensation for these low-nutrient, high-energy-density snacks, leading to higher daily energy intake when consumed [41,42].

Our findings support further research on interventions and policies that may reduce UPF consumption or improve dietary quality. Interventions and policy examples, such as nutrition education through public health messages [43], front-of-packaging warning labels [44], or sugar-sweetened beverage taxes [45], are supported by external literature but were not directly evaluated in the present study.

Our study has several strengths. An important innovation of our study was to test for the within-person association between UPF energy share and energy intake to further understand the association of UPF consumption with total food intake in a non-experimental, ad libitum setting. Most evidence on within-person assessment of food intake comes from experimental, controlled studies. While such studies have the advantage of high internal validity, it is difficult to blind participants to every aspect influencing eating behavior, including the food environment, automatic behaviors, and psychological and cognitive factors [46,47] that influence food choices. Observational studies such as ours can contribute to understanding the relationship between UPF and health outcomes by providing evidence from real food choices in the natural context of daily food consumption, an uncontrolled aspect of eating behavior rarely found in experimental study designs. Our design was built on observed dietary patterns in the study population, which primarily consist of a traditional diet composed mainly of non-UPFs, with occasional but increasing consumption of UPFs.

Our study has limitations. The instrument used to collect dietary data was the 24-h dietary recall, which could overestimate or underestimate food consumption. However, strategies to minimize such errors included pre-testing, validation, and quality control procedures applied during data collection [22,26]. Because our study relied on only two 24-h dietary recalls, the within-person estimate may be sensitive to random day-to-day variation in intake, and the ability to estimate usual dietary intake is limited. In this study, we aimed to capture the relationship between UPF consumption and total energy intake within participants on a given day by comparing two days of food consumption; estimating usual dietary intake was not a primary aim. Another limitation of the dietary data concerns misclassification of foods, such as when the method of preparation was unclear (for example, for pizzas, cakes, mixed dishes, and fruit drinks), which hampers the distinction between ready-to-eat UPFs and homemade non-UPF dishes. Uncertain cases were resolved by adopting the most common form of consumption in Brazil. When classifying mixed foods, the recipes’ main ingredient was used to determine whether the food was UPF or non-UPF. Sensitivity analyses that excluded foods with the most uncertain classification (i.e., the UPF subgroup “mixed dishes”) yielded results consistent with the primary findings. An additional limitation is that eating occasion-level measures were not explored in this study. Future research should explore potential behavioral mechanisms, such as changes in eating frequency and timing, that underlie differences in energy intake associated with UPF consumption. 

Inherent in cross-sectional data, results provide little causal information and may be confounded by other factors. We mitigated some disadvantages of cross-sectional data by using mixed models to estimate within-person effects. Although the within-person approach reduces confounding by time-invariant characteristics, it cannot control for unmeasured day-level factors such as appetite, sleep, illness, physical activity, stress, social eating occasions, food availability, special events, day order, or specific eating contexts, which may influence both UPF consumption and total energy intake on the same day. For instance, individuals’ usual level of physical activity was not measured in the BHBS. Although the body of evidence suggests that acute exercise does not influence energy intake on the same day or the days that follow for non-athlete individuals under normal conditions [48], the unmeasured level of physical activity may lead to residual confounding. Nonetheless, a previous study with the same population observed that the level of physical activity of an individual is highly associated with socio-demographic characteristics [49], which were included in our models.

## 5. Conclusions

Our findings suggest that UPF consumption is associated with higher same-day energy intake within individuals in a free-living setting. Supplementary observational analyses were consistent with the interpretation that the association between UPF energy share and total energy intake may reflect partial replacement of non-UPF with UPF, while providing limited support for explanations based on differences in food volume or energy density, highlighting the need for further research into the underlying mechanisms. Future longitudinal and experimental studies are needed to determine whether reducing UPF consumption lowers daily energy intake over time and contributes to the prevention of weight gain and other diet-related diseases.

## Figures and Tables

**Table 1:** Sociodemographic characteristics, total daily energy intake, and daily energy share of ultra-processed foods (UPFs) in the study population (Brazil, 2017–2018, N = 38,854).

		Proportion, %	Total Energy Intake, kcal	Frequency of UPF Consumers, %	UPF Energy Share, %kcal
		Mean (SE) ^a^			
Sex					
	Male	47.3 (0.3)	1961 (11)	91.6 (0.3)	20.0 (0.3)
	Female	52.7 (0.3)	1566 (8) *	93.5 (0.3) *	21.1 (0.2) *
Age group					
	Adolescent	17.7 (0.3)	1822 (15)	95.7 (0.4)	27.0 (0.4)
	Adult	63.9 (0.4)	1793 (9)	92.5 (0.3)	20.1 (0.2)
	Elder	18.4 (0.4)	1547 (14) *	89.8 (0.5) *	16.1 (0.3) *
Schooling					
	4 years or less	17.8 (0.3)	1603 (14)	84.8 (0.8)	15.1 (0.3)
	5–8 years	25.2 (0.3)	1738 (12)	91.7 (0.4)	19.1 (0.3)
	9–12 years	40.5 (0.4)	1814 (11)	94.8 (0.3)	21.8 (0.4)
	13 years or more	16.5 (0.4)	1787 (17) *	97.0 (0.3) *	25.7 (0.5) *
Geographic area					
	Rural	15.5 (0.3)	1787 (19)	86.2 (0.7)	14.5 (0.3)
	Urban	84.5 (0.3)	1747 (9)	93.8 (0.3) *	21.7 (0.2) *
Geopolitical region					
	North	8.4 (0.3)	1798 (26) ^†^	88.7 (0.9) ^†^	18.2 (0.5) ^†,‡^
	Northeast	27.4 (0.4)	1778 (13) ^†^	89.9 (0.5) ^†^	17.3 (0.2) ^†^
	Southeast	42.3 (0.5)	1700 (14)	94.2 (0.5) ^‡^	21.9 (0.4)
	South	14.5 (0.3)	1813 (22) ^†^	95.8 (0.5) ^‡^	24.7 (0.5)
	Center–West	7.4 (0.2)	1792 (23) ^†^	91.4 (0.7) ^†^	19.9 (0.5) ^‡^
Per capita income ^b^					
	First quintile	20.6 (0.6)	1651 (17)	87.9 (0.8)	17.0 (0.4)
	Second quintile	20.1 (0.5)	1748 (14)	91.0 (0.5)	18.6 (0.3)
	Third quintile	19.9 (0.5)	1762 (17)	92.8 (0.5)	20.4 (0.4)
	Fourth quintile	19.8 (0.5)	1807 (17)	94.4 (0.4)	21.9 (0.4)
	Fifth quintile	19.6 (0.6)	1801 (17) *	97.1 (0.4) *	25.2 (0.5) *

Abbreviation: UPF, ultra-processed food. ^a^ Sample size (N = 38,854) is unweighted. Percentages, means, SEs, and regression-based comparisons were weighted to account for study design and to be nationally representative. ^b^ Average monthly per capita income by quintile: first, R$334.67; second, R$722.17; third, R$1150.88; fourth, R$1799.87; fifth, R$5250.17. The currency conversion rate on 15 January 2018 was 1.00 US$ = 3.196 BR$. * Significant linear trend across all categories for the ordinal variables (*p* for trend < 0.05), or significant difference between categories for the binary variables sex and geographic area (*p* < 0.05). ^†,‡^ Values sharing the same symbol are not significantly different, for the non-ordinal variable geopolitical region (*p* ≥ 0.05).

**Table 2:** Unadjusted and adjusted mixed models testing the association between daily dietary share of ultra-processed foods (UPF) and daily energy intake (kcal) (Brazil, 2017–2018, N = 38,854).

	Unadjusted Model		Adjusted Model ^a^	
	Daily Energy Intake (kcal)			
Fixed effects ^b^	Estimate	95% CI	Estimate	95% CI
Intercept	1648.3	1637.2–1659.3	1851.5	1822.2–1880.7
UPF energy share (%kcal)				
Within-person	4.06 *	3.67–4.44	3.90*	3.50–4.29
Between-person	5.77 *	5.32–6.23	5.73*	5.28–6.18
Random effects ^c^	Mean	SE	Mean	SE
Person-level	340,088	3775	268,719	3280
Residual	321,823	2309	318,015	2284
	Estimate	95% CI	Estimate	95% CI
ICC	0.514	0.506–0.521	0.458	0.450–0.466

Abbreviations. CI: Confidence interval; UPF: ultra-processed food; ICC: Intraclass correlation coefficient. ^a^ Model adjusted for day of the week, location, sex, age group, schooling, per capita income, geographic area, and geopolitical region. ^b^ Fixed effects are estimates of the UPF dietary share components (within- and between-person). ^c^ Random effects are variance estimates. * *p* < 0.001.

## Data Availability

The 2017–2018 Brazilian Household Budget Survey data that support the findings of this study are available from the Brazilian Institute of Geography and Statistics (IBGE—Instituto Brasileiro de Geografia e Estatística) website (“POF—Consumer Expenditure Survey, Microdata”, https://www.ibge.gov.br/en/statistics/social/population/25610-pof-2017-2018-pof-en.html?=&t=microdados (accessed on May 25 2025).

## Supplementary Materials

The following supporting information can be downloaded at: https://www.mdpi.com/article/10.3390/nu18132075/s1, Figure S1: Adjusted mixed model estimates for total daily energy intake in each day according to the ultra-processed food (UPF) consumption patterns: “No UPF consumption on any day”, “UPF consumption on one day only”, or “UPF consumption on two days” (Brazil, 2017–2018, N = 38,854); Figure S2: Observed distribution of energy share of ultra-processed foods (UPFs; %kcal) by sex and age group (Brazil, 2017–2018, N = 38,854); Table S1: Classification rules for commonly ambiguous items using the Nova Classification System; Table S2: Energy and nutrient density on each day by ultra-processed food (UPF) consumption pattern (Brazil, 2017–2018, N = 38,854); Table S3: Average energy intake (kcal; mean and SD) from ultra-processed food (UPF) subgroups on the day with UPF among participants in the “UPF consumption on one day only” pattern, by sex and age group (Brazil, 2017–2018, *n* = 8055); Table S4: Descriptive statistics of the ultra-processed food (UPF) subgroups with the most uncertain classification and the within-person coefficients from adjusted mixed models testing the association between daily dietary share of UPF and daily energy intake (kcal), excluding each of the UPF subgroups (Brazil, 2017–2018, N = 38,854); Table S5: Supplementary mixed models examining the relationship between ultra-processed food (UPF), non-UPF, and total energy intake (kcal) and the association between UPF energy share (%kcal) and the alternative dietary measures energy density (g/kcal) and total amount consumed (g) (Brazil, 2017–2018, N = 38,854).

## Abbreviations

UPF: ultra-processed food; BHBS: Brazilian Household Budget Survey.
