# Supplementary material for: Within-Person Variation in Ultra-Processed Food Consumption Is Associated with Total Daily Energy Intake
Source: Nutrients. 2026 Jun 24;18(13):2075. doi: 10.3390/nu18132075 (PMC13362714; doi:10.3390/nu18132075)
Supplement: Supplementary file 1 [file nutrients-18-02075-s001.zip › Supplementary Figures Revised.pdf]

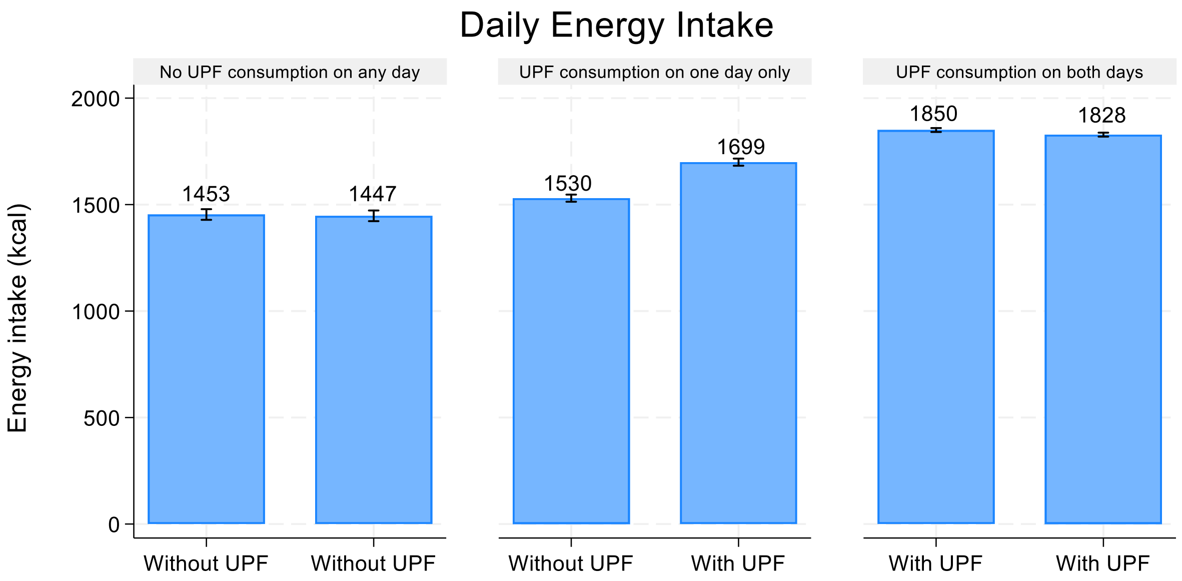

Supplementary Figure S1: Adjusted mixed model estimates for total daily energy intake in each day according to the ultra-processed food (UPF) consumption patterns: "No UPF consumption on any day", "UPF consumption on one day only", or "UPF consumption on both days" (Brazil, 2017-2018, N = 38,854). Abbreviation. UPF: ultra-processed food. Note. The unweighted sample sizes and percentages of participants in the "No UPF consumption on any day", "UPF consumption on one day only", and "UPF consumption on both days" patterns were, respectively: $n = 3669$ (9.5%), $n = 8055$ (20.7%), and $n = 27,130$ (69.8%). The mixed model was adjusted for day of the week, location, sex, age group, schooling, per capita income, geographic area, and geopolitical region. †‡+§⁑ Values sharing the same symbol are not significantly different ($p \geq 0.05$). Comparisons between days in the three UPF consumption patterns represent between-person differences in average daily energy intake. Only the "UPF consumption on one day only" pattern allows a within-person comparison between a day with UPF consumption and one without.

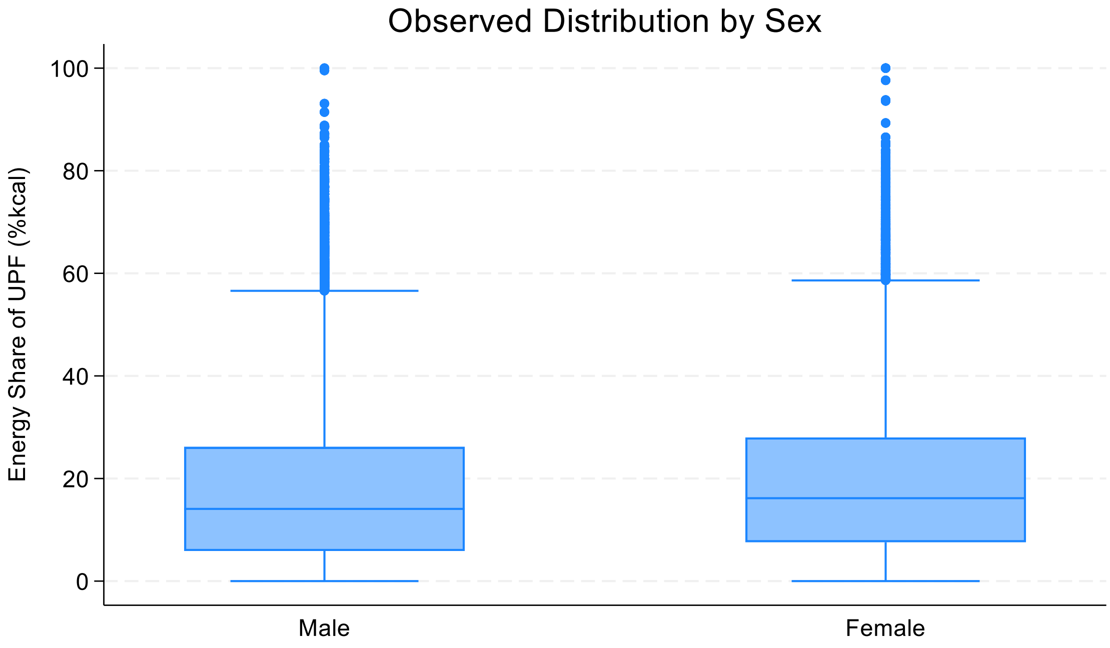

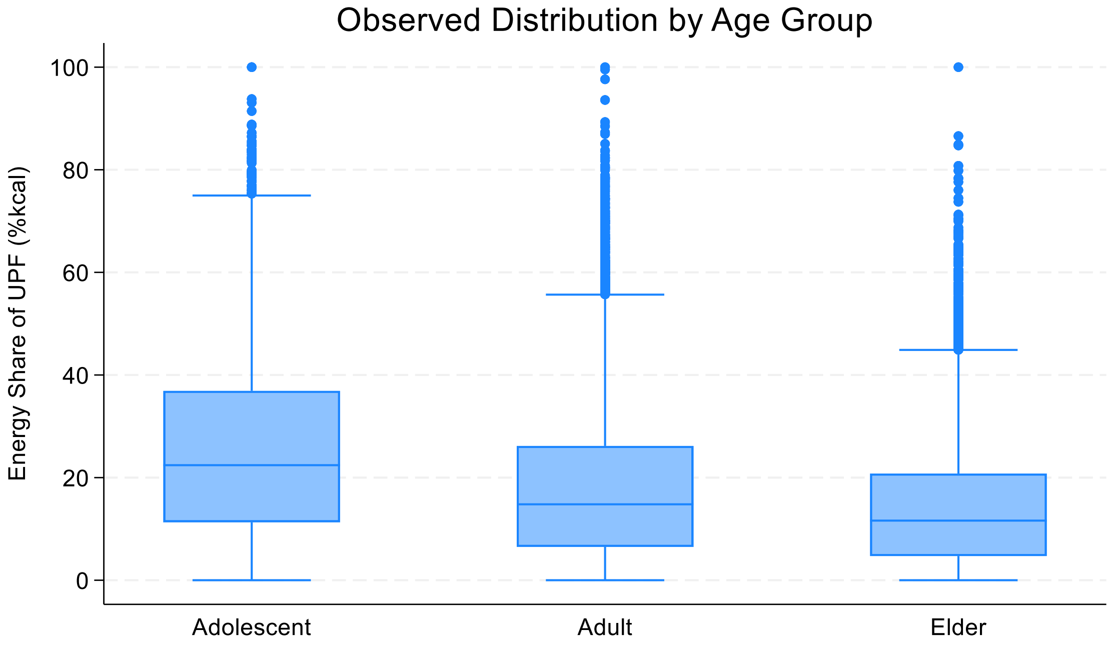

Supplementary Figure S2: Observed distribution of energy share of ultra-processed foods (UPFs; %kcal) by sex and age group (Brazil, 2017-2018, N = 38,854). Abbreviation. UPF: ultra-processed food. Note. Unweighted sample sizes: (by sex) Male, *n* = 17,969; Female, *n* = 20,885; (by age group) Adolescent, *n* = 7092; Adult, *n* = 24,508; Elder, *n* = 7254.
