# Supplementary material for: Within-Person Variation in Ultra-Processed Food Consumption Is Associated with Total Daily Energy Intake
Source: Nutrients. 2026 Jun 24;18(13):2075. doi: 10.3390/nu18132075 (PMC13362714; doi:10.3390/nu18132075)
Supplement: Supplementary file 1 [file nutrients-18-02075-s001.zip › Supplementary Tables Revised v.4.pdf]

Supplementary Table S1: Classification rules for commonly ambiguous items using the Nova Classification System.

Food item	Classification rule	Nova group(s)
Pizza	Typically industrial and ready-to-heat; assumed UPF	• UPF (subgroup "ready-to-heat pizza")
Cake	Classified by main ingredient; assumed UPF if it contained a UPF component; assumed not-UPF if homemade specified	• UPF (subgroup "cakes and pies"; e.g., chocolate cake)<br>• Non-UPF (e.g., carrot cake)
Fruit juice	Assumed industrialized and UPF unless fresh/natural specified	• UPF (subgroup "fruit drinks"; e.g., mango juice)<br>• Non-UPF (e.g., natural orange juice)
Mixed dishes	Classified by main ingredient; assumed UPF if it contained a UPF component; assumed not-UPF if homemade specified	• UPF (subgroup: "mixed dishes"; e.g., mayo salad)<br>• Non-UPF (e.g., beef stew)
Sandwiches	Classified by main ingredient; assumed UPF if main ingredient is UPF	• UPF (subgroup "ready-to-heat/eat dishes"; e.g., hot dog on a bun)<br>• Non-UPF (e.g., egg sandwich)

Abbreviation. UPF: ultra-processed food.

S**upplementary Table S2**: Energy and nutrient density on each day by ultra-processed food (UPF) consumption pattern (Brazil, 2017-2018, N = 38,854).

**Nutrient Measure**	**No UPF Consumption on Any Day**		**UPF Consumption on One Day Only**		**UPF Consumption on Both Days**	
	**Without UPF**	**Without UPF**	**Without UPF**	**With UPF**	**With UPF**	**With UPF**
	Mean (95% CI)					
Energy density (kcal/g)	1.24 (1.24; 1.25)	1.24 (1.23; 1.25)	1.24 (1.24; 1.25)	1.28 (1.27; 1.29)	1.30 (1.29; 1.30)	1.29 (1.29; 1.30)
Carbohydrate (g/1000 kcal)	135.4 (134.2; 136.5)	135.6 (134.4; 136.7)	134.0 (133.3; 134.8)	136.1 (135.4; 136.8)	136.8 (136.5; 137.1)	135.9 (135.6; 136.2)
Protein (g/1000 kcal)	53.4 (52.8; 54.0)	53.6 (53.0; 54.2)	52.3 (51.9; 52.7)	46.8 (46.4; 47.1)	44.7 (44.6; 44.9)	44.7 (44.6; 44.9)
Fat (g/1000 kcal)	30.6 (30.3; 31.0)	30.1 (29.8; 30.5)	31.1 (30.9; 31.3)	32.2 (32.0; 32.4)	32.8 (32.7; 32.9)	32.8 (32.7; 32.9)
Fiber (g/kcal)	16.6 (16.3; 16.9)	16.0 (15.7; 16.3)	14.9 (14.7; 15.0)	13.6 (13.5; 13.8)	12.8 (12.7; 12.9)	12.2 (12.2; 12.3)
Sodium (mg/1000 kcal)	1428 (1405; 1451)	1386 (1368; 1403)	1381 (1366; 1395)	1476 (1463; 1488)	1448 (1442; 1454)	1461 (1454; 1467)

Abbreviation. UPF: ultra-processed food. Note. Values are presented as unweighted mean (95% CI lower; upper). The unweighted sample sizes and percentages of participants in the "No UPF consumption on any day", "UPF consumption on one day only", and "UPF consumption on both days" patterns were, respectively: n = 3669 (9.5%), n = 8055 (20.7%), and n = 27,130 (69.8%).

Supplementary Table S3: Average energy intake (kcal; mean and SD) from ultra-processed food (UPF) subgroups on the day with UPF among participants in the "UPF consumption on one day only" pattern, by sex and age group (Brazil, 2017-2018, *n* = 8055).

Ultra-processed Food Subgroup	Examples of Foods	Sex		Age Group		
		Male	Female	Adolescent	Adult	Elder
		Energy Intake, kcal				
		Mean (SD)				
Alcoholic beverages	Cocktails; spirits; liquors	13.1 (215.2)	1.6 (43.0)	3.3 (70.2)	8.5 (179.0)	4.9 (76.9)
Bread	White bread; buns	15.7 (62.3)	17.2 (64.0)	16.2 (68.7)	16.2 (63.6)	17.4 (58.3)
Breakfast cereals	Corn cereal; granola	0.9 (27.4)	1.6 (28.9)	1.0 (20.1)	1.5 (32.8)	0.7 (12.7)
Cakes and pies	Chocolate cake; brownie	7.3 (62.5)	8.2 (56.9)	11.3 (67.8)	7.7 (61.1)	5.6 (48.1)
Cheese	Cheese spread	0.4 (5.8)	0.8 (12.9)	0.2 (5.1)	0.6 (11.4)	0.9 (8.0)
Cookies and pastries	Chocolate cookie; biscuits	29.4 (134.2)	25.9 (101.8)	46.8 (191.7)	26.2 (109.6)	19.8 (73.4)
Dairy-based drinks and yogurts	Chocolate milk; flavored yogurt	6.4 (39.8)	7.4 (37.6)	13.4 (53.7)	6.5 (37.7)	4.1 (28.4)
Dietary supplements	Nutritional powders; protein shakes	0.1 (2.7)	0.1 (2.8)	0.0 (0.0)	0.1 (2.4)	0.1 (4.2)
Energy drinks and other beverages	Ready-to-drink tea; ready-to-drink caffeinated drinks	7.6 (50.9)	7.8 (47.9)	5.3 (43.1)	7.7 (49.8)	9.4 (51.5)
Fruit drinks	Powdered fruit mix; drink mixes	11.3 (46.6)	10.2 (38.8)	13.7 (49.9)	10.6 (41.9)	9.2 (39.8)
Mixed dishes	Mayonnaise salad; rice with sausage	25.7 (159.8)	20.4 (98.3)	22.1 (101.9)	23.5 (143.8)	21.9 (102.6)
Ready-to-eat/heat dishes	Hamburger on a bun; instant noodles; ham and cheese sandwich	54.8 (188.7)	51.0 (162.6)	75.6 (224.2)	55.6 (179.2)	29.6 (115.7)
Ready-to-heat pizza	Frozen pizza	8.9 (94.8)	10.2 (103.5)	9.4 (82.3)	11.2 (111.7)	4.6 (61.3)
Reconstituted meat products	Nuggets; sausage; ham	28.7 (102.6)	18.1 (68.9)	25.4 (88.5)	24.7 (89.3)	16.9 (76.2)
Sauces, spreads, and condiments	Mayonnaise; margarine; dressing	41.7 (77.3)	32.7 (69.1)	35.2 (72.7)	39.6 (75.9)	29.9 (64.1)
Savory snacks	Crackers; chips; puffs	42.8 (144.7)	46.1 (126.8)	59.1 (150.2)	43.5 (137.1)	38.6 (119.3)
Soft drinks, carbonated	Cola soft drinks; orange-flavored soft drinks	26.0 (73.2)	17.5 (54.0)	26.8 (72.4)	23.3 (66.6)	12.6 (46.4)
Sweets	Chocolate; ice cream; cereal bar	13.4 (90.6)	15.5 (107.0)	27.0 (164.4)	12.7 (86.3)	12.2 (81.3)

Abbreviation. UPF: ultra-processed food. Note. Unweighted sample sizes: (by sex) Male, *n* = 3816; Female, *n* = 4239; (by age group) Adolescent, *n* = 1078; Adult, *n* = 5289; Elder, *n* = 1688.

Supplementary Table S4: Descriptive statistics of the ultra-processed food (UPF) subgroups with the most uncertain classification and the within-person coefficients from adjusted mixed models testing the association between daily dietary share of UPF and daily energy intake (kcal), excluding each of the UPF subgroups (Brazil, 2017-2018, N = 38,854).

	**Descriptive Statistics[a]**			**Adjusted Mixed Model[b] Within-person Fixed Effects**		
**Ultra-processed Food Subgroup**	**Energy Intake (kcal)**	**Energy Share of Total Intake (%kcal)**	**Energy Share of Total UPF Intake (%kcal)**			
	Mean (SD)			Estimate	95% CI	*p*-value
Cakes and pies	9.5 (61)	0.5 (3.2)	1.3 (9.1)	3.68	3.29 – 4.08	<0.001
Fruit drinks	9.1 (41)	0.5 (2.2)	3.4 (14.6)	3.92	3.52 – 4.21	<0.001
Mixed dishes	24.8 (123)	1.3 (5.9)	3.8 (16.0)	3.62	3.22 – 4.03	<0.001
Ready-to-heat pizza	9.7 (103)	0.4 (4.1)	0.9 (7.7)	3.21	2.81 – 3.60	<0.001

[a] The estimates of the descriptive statistics for ultra-processed food subgroups are unweighted and calculated for the total sample N = 38,854. [b] The mixed models were adjusted for day of the week, location, sex, age group, schooling, per capita income, geographic area, and geopolitical region.

Supplementary Table S5: Supplementary mixed models examining the relationship between ultra-processed food (UPF), non-UPF, and total energy intake (kcal) and the association between UPF energy share (%kcal) and the alternative dietary measures energy density (g/kcal) and total amount consumed (g) (Brazil, 2017-2018, N = 38,854).

**Supplementary model**	**Dependent variable**	**Independent variable**	**Adjusted mixed model[a] Within-person fixed effects**		
			Estimate	95% CI	*p*-value
Model 1	Total energy intake (kcal)	UPF energy intake (kcal)	0.763	0.748 – 0.778	<0.001
Model 2	Total energy intake (kcal)	Non-UPF energy intake (kcal)	0.889	0.882 – 0.896	<0.001
Model 3	Non-UPF energy intake (kcal)	UPF energy share (%kcal)	-0.237	−0.252 – −0.222	<0.001
Model 4	Energy density (g/kcal)	UPF energy share (%kcal)	0.003	0.002 – 0.003	<0.001
Model 5	Total amount consumed (g)	UPF energy share (%kcal)	-0.134	−0.490 – 0.223	0.462

Abbreviation. CI: Confidence interval. UPF: ultra-processed food. [a] Models were adjusted for day of the week, location, sex, age group, schooling, per capita income, geographic area, and geopolitical region. Note. Unweighted mean, standard error, and 95% CI of variables in the models: total energy intake, 1756 kcal, SE = 2.9, 95% CI 1750 to 1762; UPF energy intake, 346 kcal, SE = 1.6, 95% CI 343 to 349; non-UPF energy intake, 1410 kcal, SE = 2.6, 95% CI 1405 to 1415; UPF energy share, 18.7 %kcal, SE = 0.1, 95% CI 18.5 to 18.8; energy density, 1.28 g/kcal, SE ~ 0.0, 95% CI 1.28 to 1.29; total food consumed, 1449 g, SE = 2.8, 95% CI 1443 to 1454.
